# *Chaerilus pseudoconchiformus* sp. n. and an updated key of the chaerilid scorpions from China (Scorpiones, Chaerilidae)

**DOI:** 10.3897/zookeys.495.9016

**Published:** 2015-04-08

**Authors:** Shijin Yin, Yanning Qiu, Zhaohui Pan, Shaobin Li, Zhiyong Di

**Affiliations:** 1 College of pharmacy, South Central University for Nationalities, Wuhan 430074, P.R. China; 2 School of Life Sciences, University of Science and Technology of China, Hefei 230027, P.R. China; 3 Institute of Plateau Ecology, Agriculture and Animal Husbandry College of Tibet University, Linzhi 860000, P.R. China; 4 College of Life Sciences, Yangtze University, Jingzhou 434025, P.R. China

**Keywords:** Chaerilidae, *Chaerilus*, new species, Xizang, China

## Abstract

A new species, *Chaerilus pseudoconchiformus* **sp. n.**, is described from Xizang, China. The present new species is distinguished from its congeners by a body length of 32−40 mm, carapace with the anterior margin straight, chela with length/width ratio average of 3.3 in males (3.2−3.4, two adults), and 2.5 in females (2.3−2.6, nine adults), eight or nine (eight usually) rows of denticles on fixed and movable fingers of pedipalp chelae, five pectinal teeth in males and three or four in females. To date, the chaerilid species fauna of China consists of nine species. An updated identification key to *Chaerilus* from China is presented.

## Introduction

The small monotypic family, Chaerilidae, has been reported containing one genus with 39 species (1/2015, http://www.ntnu.no/ub/scorpion-files/). The only genus is *Chaerilus*, which is found in southern and southeast Asia. In Xizang (Tibet), the chaerilid scorpions live under stones and fallen trees in humid habitats.

Chaerilid scorpions have a unique type B trichobothrial arrangement (Vachon 1974; Soleglad and Fet 2001). Kovařík (2000) reported 18 species in this genus in his review. Kovařík (2012) published an identification key for the genus. Recently, new species were described (Kovařík et al. 2014; Lourenço and Pham 2014).

Kovařík (2000) reported an old species and erected a new species of chaerilid from Xizang in his revision: *Chaerilus pictus* (Pocock, 1890) and *Chaerilus tryznai* Kovařík, 2000. In fact, one locality of *Chaerilus tricostatus* Pocock, 1899, Upper Rotung (Abor District), is also a territory belonging to Xizang (China). Therefore, Kovařík’s revision recorded three species for China (Di et al. 2009). Zhu et al. (2004) recorded one chaerilid species (*Chaerilus pictus*) found in China. Qi et al. (2005) described one new species (*Chaerilus tessellatus* Qi, Zhu & Lourenço, 2005) and redescribed *Chaerilus pictus* (misidentification). Bastawade (2006) reported a new species from southeast Xizang: *Chaerilus dibangvalleycus* Bastawade, 2006. Zhu et al. (2008) redescribed *Chaerilus tessellatus* and *Chaerilus tryznai*, and pointed out that *Chaerilus pictus* as redescribed by Qi et al. (2005) was misidentified and erected it as a new species: *Chaerilus conchiformus* Zhu, Han & Lourenço, 2008. Zhu et al. (2008) also suggested that distribution of *Chaerilus pictus* in China was doubtful. Di and Zhu (2009) reported one new species: *Chaerilus mainlingensis* Di & Zhu, 2009. Di et al. (2009) reviewed the genus *Chaerilus* in China, registered seven species, and described the female of *Chaerilus tricostatus* for the first time. Kovařík (2012) described a new species from Xizang: *Chaerilus wrzecionkoi* Kovařík, 2012. Di et al. (2014) reviewed the research history of the order Scorpiones from China, and recorded eight chaerilid species. To date, the chaerilid fauna of China consists of nine species including the new species described in this paper, *Chaerilus pseudoconchiformus* sp. n.

## Material and methods

Illustrations and measurements were made using a Motic K700 stereomicroscope with an Abbe drawing tube and an ocular micrometer. The photos were taken with a Canon (650D) camera. Measurements follow Sissom (1990) and are given in mm. Trichobothrial notations follow Vachon (1974) and morphological terminology mostly follows Hjelle (1990). Research materials have been deposited in the Specimen Room of University of Science and Technology of China, Hefei, China (USTC).

## Taxonomy

### Family Chaerilidae Pocock, 1893 Genus *Chaerilus* Simon, 1877

#### Chaerilus pseudoconchiformus sp. n.

Taxon classificationAnimaliaScorpionesChaerilidae

http://zoobank.org/569B23FC-86FB-4E16-8487-047B100BF9DC

Figs 1–4 , 5−25 , 26–35 , Tables 1 , 2

##### Type material.

Holotype, male, China: Xizang, Nyingchi County (Linzhi County), VIII/2014, Zhiyong Di and Tao Li leg. (Ar.-USTC-XZLZ1401); paratypes: 1 adult male, 9 adult females, same data as holotype (Ar.-USTC-XZLZ1402−11) (kept in USTC).

##### Diagnosis.

The new species differs from its congeners by the following features: approximately 30−40 mm in total length (Table 2); carapace with the anterior margin straight; chela with length/width ratio: average of 3.3 in males, and 2.5 in females (Table 2); eight or nine (usually eight) rows of denticles on fixed and movable fingers of pedipalp chelae; five pectinal teeth in males and three or four in females. *Chaerilus pseudoconchiformus* sp. n. can be distinguished from the geographically and morphologically closely related species (Tables 2–3, and key). Morphologically closest are *Chaerilus conchiformus* and *Chaerilus wrzecionkoi*. Both these species have similar body lengths, as well as similar numbers of denticle rows on fixed and movable fingers of the pedipalp chelae. They can be distinguished by the length/width ratio of the pedipalp chela: manus of pedipalp in male narrow and long, chela length/width ratio in male higher than 3 (average of 3.3 in two males, and 2.5 in nine females) in *Chaerilus pseudoconchiformus* sp. n.; manus of pedipalp in male robust (Kovařík 2012: Fig. 68), chela length/width ratio in both sex adults lower than 2.6 in *Chaerilus wrzecionkoi* (Kovařík, 2012: 2); manus of pedipalp in both sex adults robust (Zhu et al. (2008): Figs 3, 17), chela length/width ratio in one male adult is 2.4 (paratype: Ar.–MHU–XZ0102), in two females (including the holotype) lower than 2.0 in *Chaerilus conchiformus*. Furthermore, *Chaerilus pseudoconchiformus* sp. n. has more slender pedipalps than *Chaerilus wrzecionkoi* (Table 1; Kovařík 2012: 13), in other words, the length ratio of pedipalp (LRP) is distinctly larger than the length ratio of total length (LRT) of *Chaerilus pseudoconchiformus* sp. n. and *Chaerilus wrzecionkoi*: 1.14 (LRP), 1.01 (LRT) in male holotypes; 1.08 (LRP), 0.95 (LRT) in female allotypes of *Chaerilus pseudoconchiformus* sp. n. and *Chaerilus wrzecionkoi* (Table 1).

**Figures 1–4.**
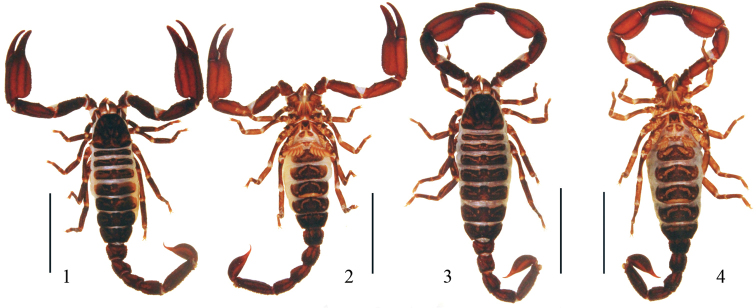
*Chaerilus pseudoconchiformus* sp. n., dorsal and ventral habitus: **1–2** Male holotype (Ar.−USTC−XZLZ1401) **3–4** Female paratype (Ar.−USTC−XZLZ1402). Scale bar = 10 mm.

**Figures 5–25.**
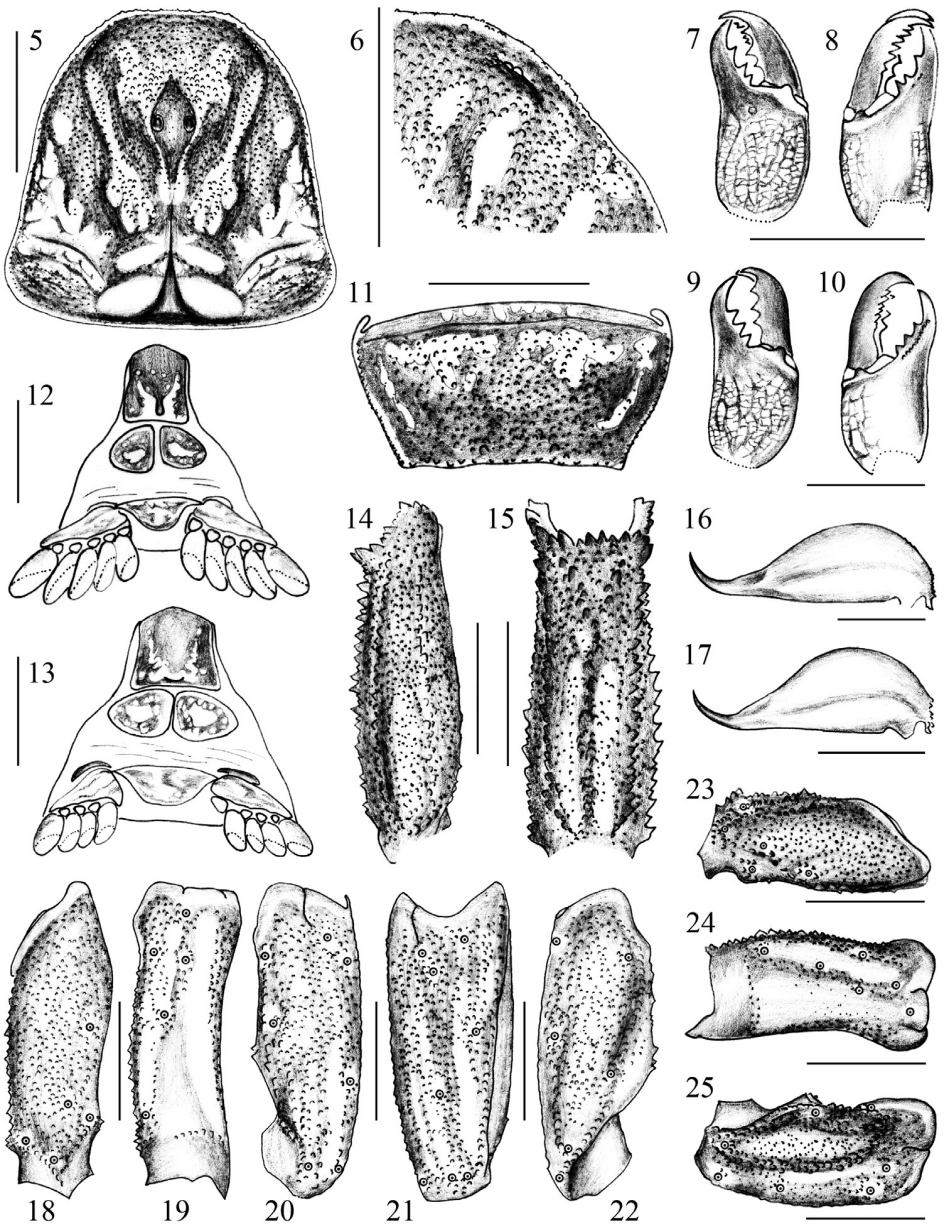
*Chaerilus pseudoconchiformus* sp. n. Male holotype: **5** Carapace, dorsal aspect **6** Lateral eyes area **7–8** Chelicera, dorsal and ventral aspects **11** Tegument of the seventh sternite; **12** Sternum, genital operculum and pectines **14–15** Metasomal segment V, lateral and ventral aspects **16** Telson **18–19** Femur, dorsal and external aspects **20–22** Patella, dorsal, external and ventral aspects. Female paratype (Ar.−USTC−XZLZ1402): **9–10** Chelicera, dorsal and ventral aspects **17** Telson **23–24** Femur, dorsal and external aspects **25** Patella, dorsal aspect. Scale bars = 2 mm.

**Table 1.** Measurements (mm) of *Chaerilus pseudoconchiformus* sp. n., male holotype (Ar.-USTC-XZLZ1401) and female paratype (Ar.-USTC-XZLZ1402). The information of *Chaerilus wrzecionkoi* from Kovařík (2012).

	*Chaerilus pseudoconchiformus* sp. n.		*Chaerilus wrzecionkoi*	
	Male holotype	Female paratype	Male holotype	Female paratype
Total length	37.4	37.1	37.0	39.0
Carapace: -Length -Anterior width -Posterior width	4.5 2.4 5.0	4.4 2.7 5.3	4.3 4.4	4.5 5.1
Mesosomal segments: -Length	11.3	13.5		
Metasomal segment I: -Length -Width -Depth	2.0 2.9 2.1	1.8 2.8 2.1	2.0 2.4	1.8 2.7
Metasomal segment II: -Length -Width -Depth	2.6 2.4 1.9	2.2 2.4 1.8	2.4 2.0	2.2 2.2
Metasomal segment III: -Length -Width -Depth	2.9 2.3 1.7	2.5 2.2 1.8	2.4 2.0	2.2 2.0
Metasomal segment IV: -Length -Width -Depth	3.3 2.1 1.6	3.0 2.0 1.6	2.7 1.9	2.7 1.8
Metasomal segment V: -Length -Width -Depth	5.4 1.9 1.5	4.9 1.8 1.5	4.7 1.8	4.4 1.6
Telson: -Length -Width -Depth	5.5 2.1 1.7	4.8 2.0 1.7	4.9	4.9
Pedipalp femur: -Length -Width -Depth	5.4 1.8 1.9	4.1 1.7 1.9	4.5 1.6	3.7 1.7
Pedipalp patella: -Length -Width -Depth	5.3 1.9 2.1	4.1 2.0 2.4	4.8 1.7	4.0 2.2
Chela: -Length -Width (manus) -Depth (manus)	10.2 3.2 3.1	9.0 3.6 3.1	9.0 3.5	8.3 3.5
Movable finger: -Length	5.2	5.2	5.0	4.5
Pectinal teeth (left/right)	5/5	4/4	4/5	?

**Table 2.** Feature datasets of body length (BL, mm; segment by segment was measured and added in type specimens, while others were measured for overall length only), chela with length/width ratio (CR), number of granule rows of movable finger of pedipalp (RN), and number of pectinal teeth (PT) of *Chaerilus conchiformus* (CO, Ar.-USTC-XZLZ1412), *Chaerilus pseudoconchiformus* sp. n., and *Chaerilus tryznai* (TY, Ar.-USTC-XZBM1401−02).

	Sex	BL	CR	RN	PT
XZLZ1401	♂	37.4	3.2	8/8	5/5
XZLZ1402	♀	37.1	2.5	8/8	4/4
XZLZ1403	♂	32.0	3.4	8/8	5/5
XZLZ1404	♀	36.0	2.5	8/8	4/4
XZLZ1405	♀	39.0	2.6	9/9	4/4
XZLZ1406	♀	32.5	2.4	8/8	3/3
XZLZ1407	♀	38.0	2.6	8/8	4/3
XZLZ1408	♀	37.0	2.3	8/8	3/3
XZLZ1409	♀	38.0	2.3	8/8	3/3
XZLZ1410	♀	37.0	2.5	8/8	4/3
XZLZ1411	♀	35.5	2.5	8/8	4/3
XZLZ1412(CO)	♀	32.0	1.9	7/7	4/4
XZBM1401(TY)	♀	44.0	2.8	8/8	3/3
XZBM1402(TY)	♀	39.0	2.6	8/8	3/3

**Table 3.** The differences between chaerilids from China: *Chaerilus conchiformus*, *Chaerilus dibangvalleycus*, *Chaerilus mainlingensis*, *Chaerilus pictus*, *Chaerilus pseudoconchiformus* sp. n., *Chaerilus wrzecionkoi*, *Chaerilus tessellatus*, *Chaerilus tricostatus*, and *Chaerilus tryznai*; body length (BL, mm); carapace with the anterior margin (straight or curving, CA); chela with length/width ratio respectively in females and males (CR(F), CR(M)), dorsal secondary carinae of the chela (DS); rows number of denticles on fixed and movable fingers of chelae (RF); the tegument of the seventh sternite (SVII); holotype (H), paratype (P), new material (N).

	*conchiformus* (H&N)	*dibangvalleycus* (H&P)	*mainlingensis* (H&P)	*pictus* (H&P)	*pseudoconchiformus* sp. n. (H&P)	*wrzecionkoi* (H&P)	*tessellatus* (H&P)	*tricostatus* (N)	*tryznai* (H, P&N)
BL	32−44	36−42	40−41	38−66	32–39	33−41	35−52	48−60	30–44
CA	straight	slightly curving	slightly depressed	slightly curving	straight	straight	straight	straight	straight
CR(F)	1.8−1.9	?	2.4−2.8	2.4	2.3−2.6	2.4	2.2	2.2−2.4	2.6−2.9
CR(M)	?	?	?	2.5	3.2−3.4	2.6	?	3.7	>3
DS	present	absent	absent	present	present	present	present	absent	present
RF	8	7 or 8 *^1^	7	13 or 14	8 or 9	8(9?) *^2^	11	11or 12	8
SVII	weakly granular; with carinae	granular; with carinae	weakly granular; with carinae	?	granular; without carinae	granular; without carinae	with carinae	granular; with carinae	granular; without carinae

*^1^ Nine rows of denticles on fixed and movable fingers of pedipalp chelae in the holotype (Bastawade 2006: Fig. 5). But the author thought that there are seven or eight rows in *Chaerilus dibangvalleycus* and 10−11 in *Chaerilus tricostatus* (Bastawade 2006: 454).

*^2^ Nine rows of denticles on movable fingers of pedipalp chelae in the holotype (Kovařík 2012: Fig. 64), but the author described eight rows in *Chaerilus wrzecionkoi* (Kovařík 2012: 11).

##### Etymology.

The specific name refers to the geographically and morphologically most closely related species *Chaerilus conchiformus*, adding the Greek prefix “pseudo−” as “pseudoconchiformus”, because the habitus of both sexes is very similar to that of *Chaerilus conchiformus*.

##### Description.

Based on male holotype and female paratype.

*Coloration* (Figs 1–4). Basically reddish brown. Carapace dark red-brown with black parts and yellowish stripes. Mesosomal tergites dark red-brown with yellowish stripes. Metasoma: all segments dark red-brown. Telson dark red-brown with reddish brown part; aculeus light red-brown. Chelicerae reddish brown with dark reticular pattern on dorsal surface. Pedipalps: femur, patella and chela dark red-brown with dark carinae. Legs dark red-brown and red-brown on distal segments. Sternum, genital operculum and sternites red-brown with some light parts. Pectines light yellow.

*Morphology*. Carapace carinated, with the anterior margin straight; with dense granules of nearly equal size; lateral furrow moderately deep; large granules form 2 longitudinal lateral carinae (Fig. 5). Median ocular tubercle with granules. Lateral ocular tubercle small with a pair of lateral eyes and some granules (Fig. 6). Lateral eyes distinctly smaller than median eyes (Fig. 5).

*Mesosoma*: Tergites uniform distributing with granules of larger and unequal size; tergites I to II without carinae, each of tergites III to VI bearing a pairs of obsolete granular carinae on posterior margin, tergite VII bearing two pairs of obsolete granular lateral carinae, but middle pair is represented only by ridges without expressed carinae; sternum pentagonal; genital operculum triangular; pectinal teeth count 5/5 in males and 3−4 in females, with fulcra well developed (Figs 12–13); sternites III to VI are smooth, sternite VII granular without carinae (Fig. 11).

*Metasoma*: Length about 4.8 times as long as carapace in males and 4.4 in females; segment I always wider than long; segments I to V with 10-8-8-8-7 granular carinae; the ventromedian and ventrolateral carinae of segment V composed of strong, dentated granules, ventromedian carina posteriorly bifurcated as “Y” (Figs 14–15); all segments with sparse small granules. Vesicle is almost smooth; aculeus slightly curved (Figs 16–17).

*Chelicerae*: Tibia surfaces smooth; thickly covered with numerous short, silky hairs, extending to ventral aspect of chelicerae and dorsal aspect of fixed fingers; ventral inner edges of movable finger with some minute teeth (2−3 obsolete teeth in two males and 3−9 well developed and obsolete teeth in nine females) (Figs 7–10).

*Pedipalp*: Tegument granular. The femur has four carinae and the patella has five granular carinae (Figs 18–27). Chela with length/width ratio average of 3.3 in males (two adults) and 2.5 in females (nine adults), has seven granulated dorsointernal, except internal carina obsolete; entire tegument of chela manus densely covered with coarse granules, forming some indistinct reticular pattern (Figs 28–33); fingers straight, the cutting edge of movable finger with 8 or 9 (mainly 8) rows of denticles (Figs 34–35). Trichobothriotaxy of type B; orthobothriotaxic (Vachon 1974) (Figs 18–33).

**Figures 26–35.**
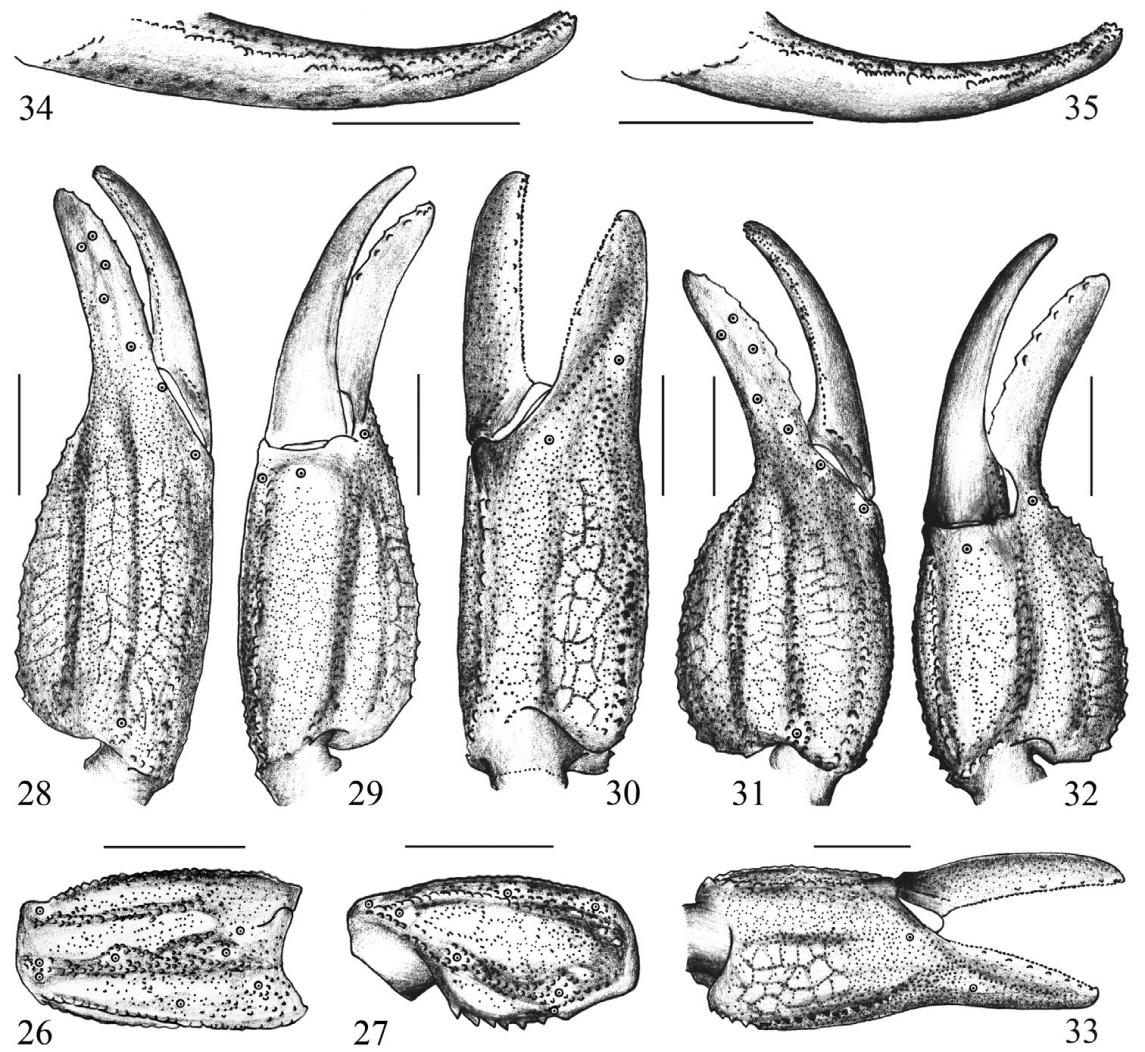
*Chaerilus pseudoconchiformus* sp. n. Male holotype: **28–30** Chela, dorsoexternal, ventral, and internal aspects **34** Movable finger of pedipalp. Female paratype (Ar.−USTC−XZLZ1402): **26–27** Patella, external and ventral aspects **31–33** Chela, dorsoexternal, ventral, and internal aspects **35** Movable finger of pedipalp. Scale bars = 2 mm.

*Legs*: Tibia without tibial spur. Basitarsus with two pedal spurs strongly developed. Tarsi with two rows of spiniform setae.

##### Variation.

Coloration and morphology in holotype and paratypes are very similar (feature datasets please see Table 2).

##### Habitat.

Found under the stones in mixed forest.

##### Distribution.

China (Xizang).

### Update key to species of the genus *Chaerilus* in China

**Table:** 

1	Movable finger of pedipalp with 7–9 rows of granules	**2**
–	Movable finger of pedipalp with 10–14 rows of granules	**7**
2	Chela length to width ratio in female adults 1.6–1.9	***Chaerilus conchiformus***
–	Chela length to width ratio in female adults higher than 2.0	**3**
3	Ventral side of seventh mesosomal segment with 2 pairs of granular carinae; carapace with anterior margin straight with a median notch	**4**
–	Ventral side of seventh mesosomal segment with many granules but without carinae; carapace with anterior margin straight without median notch	**5**
4	Pedipalp femur shorter than carapace; 8–9 minute teeth on inner ventral margins of movable and immovable fingers respectively (Bastawade 2006: 451, fig. 5)	***Chaerilus dibangvalleycus***
–	Pedipalp femur longer than carapace, 7–8 minute teeth on inner ventral margins of movable and immovable fingers respectively (Di and Zhu 2009: 101, fig. 11)	***Chaerilus mainlingensis***
5	Manus of pedipalp narrower and longer with the ventral margin not round in females (Zhu et al. 2008: fig. 47); chela length/width ratio in females is 2.6–2.9 (Kovařík 2000: table 1)	***Chaerilus tryznai***
–	Manus of pedipalp robust in females with the ventral margin very round in females; chela length/width ratio in females is 2.3–2.6	**6**
6	Chela length/width ratio in males average of 3.3 (3.2−3.4), and 2.5 in females (2.3−2.6), chelae of male and female with sexual dimorphism	***Chaerilus pseudoconchiformus* sp. n.**
–	Chela length/width ratio about 2.6 in male, and about 2.4 in female, chelae of male and female without sexual dimorphism (Kovařík 2012: 13, figs 62, 76)	***Chaerilus wrzecionkoi***
7	Movable finger of pedipalp with 13–14 rows of granules; telson of male rather long and about 4.7 times longer than wide, with an obvious sexual dimorphism in both sexes	***Chaerilus pictus***
–	Movable finger of pedipalp with 11–12 rows of granules; telsons of male and female without sexual dimorphism	**8**
8	Carapace and tergites nearly smooth in adults (Zhu et al. 2008: 44, 47)	***Chaerilus tessellatus***
–	Carapace and tergites with many big granules in adults (Di et al. 2009: 133, 136)	***Chaerilus tricostatus***

## Supplementary Material

XML Treatment for Chaerilus pseudoconchiformus
